# Effectiveness of hypofractionated and normofractionated radiotherapy in a triple‐negative breast cancer model

**DOI:** 10.3389/fonc.2022.852694

**Published:** 2022-10-27

**Authors:** Sinja Grosche, Natalia V. Bogdanova, Dhanya Ramachandran, Marcus Lüdeking, Katharina Konzok, Hans Christiansen, Christoph Henkenberens, Roland Merten

**Affiliations:** ^1^ Radiation Oncology Research Unit, Hannover Medical School, Hannover, Germany; ^2^ Gynaecology Research Unit, Medical School, Hannover, Germany; ^3^ Radiation Oncology, Hannover Medical School, Hannover, Germany; ^4^ Radiation Oncology, Dorothea Christiane Erxleben Clinic, Wernigerode, Germany

**Keywords:** hypofractionated radiotherapy, breast cancer, cell culture model, irradiation, side effects

## Abstract

Breast cancer (BC) is one of the most diagnosed malignant carcinomas in women with a triple-negative breast cancer (TNBC) phenotype being correlated with poorer prognosis. Fractionated radiotherapy (RT) is a central component of breast cancer management, especially after breast conserving surgery and is increasingly important for TNBC subtype prognosis. In recent years, moderately hypofractionated radiation schedules are established as a standard of care, but many professionals remain skeptical and are concerned about their efficiency and side effects. In the present study, two different triple-negative breast cancer cell lines, a non-malignant breast epithelial cell line and fibroblasts, were irradiated daily under normofractionated and hypofractionated schedules to evaluate the impact of different irradiation regimens on radiation-induced cell-biological effects. During the series of radiotherapy, proliferation, growth rate, double-strand DNA break-repair (DDR), cellular senescence, and cell survival were measured. Investigated normal and cancer cells differed in their responses and receptivity to different irradiation regimens, indicating cell line/cell type specificity of the effect. At the end of both therapy concepts, normal and malignant cells reach almost the same endpoint of cell count and proliferation inhibition, confirming the clinical observations in the follow-up at the cellular level. These result in cell lines closely replicating the irradiation schedules in clinical practice and, to some extent, contributing to the understanding of growth rate or remission of tumors and the development of fibrosis.

## Introduction

Breast cancer (BC) is one of the most diagnosed malignant carcinomas in women. It causes 23% of all reported cancer cases, being, furthermore, the leading cause of death among all cancer entities in women (at 14%) (1). Among all BC incidences, up to 20% account for triple‐negative breast cancer (TNBC) (2). TNBC is a heterogeneous disease often characterized by more aggressive biology than the other BC subtypes and is associated with an early age at diagnosis, larger tumor sizes, higher local-regional rates of recurrence, and *BRCA1* mutations (3–6). Concerning treatment outcome, the TNBC phenotype is correlated with poorer prognosis and is often associated with distant metastases (3). In terms of patient management, the lack of hormonal or targeted therapy and gaps in knowledge on the importance and role of radiotherapy in TNBC make this BC subtype a challenge for clinicians. Radiotherapy for breast cancer can reduce the risk of a local relapse and decrease the risk of cancer-associated mortality in the patient and is therefore a crucial part of therapeutic options for the patient (7–9). While the benefit of radiotherapy concerning overall survival for patients with TNBC is still debatable (10, 11), adjuvant radiotherapy is an indispensable part of breast conserving therapy assuring locoregional control. Thus, estimating the role of radiotherapy and the modalities of postoperative irradiation in the TNBC prognosis is continuously important. Therapy regimens can be classified into different fractionation schemes varying in duration and single doses applied. In normofractionated radiotherapy (NormRT), a total dose of usually 50 Gy is divided into single doses of 2 Gy over a longer time period resulting in 25 fractions. For hypofractionated radiotherapy (HypoRT), single doses of 2.67 Gy are applied over a shorter time period, therefore in fewer fractions, usually 15, with a total dose of about 40 Gy. Although there have been studies that show the equality of HypoRT and NormRT (12, 13), many professionals remain skeptical and concerned about the side effects of the HypoRT irradiation. Therefore, it is intensely discussed how the differently fractionated regimens of radiotherapy affect the outcome both in efficiency and toxicity. Some follow-up studies and meta-analyses have shown that the outcome of HypoRT could be compared to NormRT in efficiency, while other studies assume toxicity and side effects as fibrosis (14–18). HypoRT could potentially increase the patient’s satisfaction and compliance by reducing the amount of treatments needed while reducing costs for the health care system for shorter therapeutic periods and therefore allowing the treatment of more patients (19, 20).

We aimed to assess the effectiveness of HypoRT in comparison to NormRT in reducing the total amount of tumor cells and to compare their possible side effects and toxicity on the healthy breast tissue by investigating how normal breast epithelial cells’ and TNBC cells’ behavior is affected by both irradiation protocols. The main objective of this study was to evaluate the effects of HypoRT and NormRT on the cell proliferation capacity, cell survival, and double strand break (DSB) repair in a human breast cell model. A secondary objective was to investigate how radiation-induced effects may vary in dependence of dose per fraction and radiation duration in fibroblasts (which are under high risk for developing radiation side effects, i.e., fibrosis), during breast cancer radiotherapy. We therefore monitored radiation-induced effects *via* different approaches, during HypoRT and NormRT in different cell models.

## Materials and methods

### Cell culture

We employed the reference breast epithelial cell line MCF10A as a model for the non-malignant breast epithelium and two triple-negative BC cell lines HCC1395 and HCC1937. Both are *BRCA1*-mutant lines, with HCC1395 carrying an additional mutation in NBN (21). As an ancillary tissue type, Bj5Ta fibroblasts from a healthy donor were used. All cell lines were obtained from the American Type Culture Collection (ATCC). Cells below the passage number of 30 were taken for experiments. In all experiments, asynchronous exponentially growing cells were used. MCF10A cells were cultured in MEBM (Mammary Epithelial Cell Growth Basal Medium), supplemented with MEGM™ Single Quots™ according to the manufacturer’s instructions (Lonza). Breast cancer epithelial cell lines HCC1395 and HCC1937 were cultured in RPMI 1640 with 10% fetal calf serum (FCS), 500 U/ml penicillin, 0.5 mg/ml streptomycin, and 2 mM L-Glutamine. Bj5Ta fibroblasts were cultured in DMEM (Dulbecco′s Modified Eagle′s Medium) supplemented with 10% FCS, 500 U/ml penicillin, 0.5 mg/ml streptomycin, and 2 mM L-Glutamine. All cells were grown at 37°C in a humidified atmosphere supplemented with 5% CO_2_. After each irradiation round cells were kept and further cultured in order to undergo subsequent irradiations until the total dose for HypoRT or NormRT was achieved. For every cell line, a fixed number of cells (5 × 10^5^ for MCF10A with a doubling time of 24–36 h, and 9 × 10^5^ for all the other cell lines with a doubling time of 36 h) were seeded in 75 cm^2^ flasks 48 h before the first experimental irradiation. The medium was changed every day to remove dead cells.

### X-Ray irradiation experimental timeline

Irradiation at a dose of 2 Gy (NormRT) or 2.67 Gy (HypoRT) per fraction was applied to all the cell lines using a Synergy™ linear accelerator (Elekta AB, Stockholm, Sweden). In order to achieve all irradiation modalities for the cells comparable to the clinical setting, irradiation was carried out at about 37°C, with cells kept warm by warm pads. The dose/rate was 535 MU/min, field size 40 × 40 cm, distance 110 cm, 227 MU for NormRT and 303 MU for HypoRT. Untreated values were included in each experimental setting in such a way that for every cell line investigated, an age-matched control was incorporated. Every third irradiation day, cells were trypsinized and taken for proliferation analysis by directly counting the cell numbers and MTT assay. On irradiation days 3, 9 and 15, immunocytochemistry was performed. On irradiation day 15 (for both irradiation regimens) and on irradiation day 25 (NormRT only), the colony formation assay (CFA) and DNA synthesis-based cell proliferation (EdU incorporation) assay were performed. The senescence-associated beta-galactosidase (SA-β-gal) assay was conducted only for MCF10A and Bj5Ta cells.

### Proliferation and growth rate

To assess cellular proliferation, different methods were employed. Direct counting of cell numbers was performed manually in a Neubauer improved hemocytometer chamber and in parallel, using the Invitrogen™ Countess™ automated cell counter to exclude any observer bias. The results from manual and automated counting approaches exhibited very high similarity and were not statistically different. Cells were trypsinized as usual and resuspended in 1–5 ml of appropriate culturing media. For the statistically optimal use of the counting process, a double sampling for the manual and automated methods was performed. For this, two independent samples were taken from the cell suspension and were separately filled into the two counting areas on the counting chamber or counting slide, respectively. The average cell number from two independent values was calculated. Manual and automated counts were combined in the evaluation as technical duplicates.

To measure the cytotoxicity or growth inhibition of both irradiation regimens, the growth rate of the cells was measured by the commonly used MTT proliferation assay. This colorimetric method is based on the reduction of (3-(4,5-dimethylthiazol-2-yl)-2,5-diphenyltetrazolium bromide or MTT) to formazan crystals by metabolically active cells and is an indicator of cell viability. Briefly, every third irradiation day, 3000 cells in 100 μl of appropriate culture media per well were seeded in quadruplicates in flat-bottomed 96-well plates. Three types of the controls were used: 1) background control – wells with culture medium without cells; 2) negative control – not metabolically active cells (dead cells); and 3) positive control – all viable cells. Age-matched untreated cells were used as a positive control and as a negative control (cells were treated with 0.1% triton). After seeding, cells were incubated for 40–48 h at +37°C and 5% CO_2_. In the negative control wells, the medium was changed to a 100 µl appropriate medium, containing 0.1% triton and after 0.5 h incubation at +37°C and 5% CO_2,_ 10 μl of the MTT labeling reagent (final concentration 0.5 mg/ml) was added to each well; 96-well plates were incubated for 4 h in a humidified atmosphere (+37°C, 5% CO_2_). Culturing media were removed carefully from all wells and 100 μl of the solubilization solution (DMSO) was added into each well. The plate was covered with tinfoil and mixed in an orbital shaker for 15 min. Complete solubilization of the purple formazan crystals, which resulted in a colored solution, was checked by eye and the absorbance of the samples was measured using a microplate reader (Multiskan™ FC) at a wavelength of 540 nm. The reference wavelength was 660 nm. The average values from quadruplicate readings were determined and the average value for the blank was subtracted. The absorbance of the experimental samples was plotted on the y-axis versus the experimental day on the x-axis and compared to age-matched untreated control cultures.

DNA synthesis-based cell proliferation was measured in cells at irradiation day 15 (NormRT and HypoRT) by 5-ethynyl-2′-deoxyuridine (EdU) incorporation into newly synthesized DNA and its recognition by azide dyes *via* a copper mediated “click” reaction, using the Click-iT^®^ EdU Imaging Kit (Invitrogen). Briefly, cells were seeded on cover glasses in sterile non-coated six-well plates and incubated with 10 mM of EdU for 6–8 h. The cells were then fixed with 3.7% paraformaldehyde, EdU detection was carried out according to the manufacturer’s instructions, and nuclei were stained with Hoechst 33342 for the following analysis. For the detection of cells with replicating DNA, Alexa Fluor^®^ 488 labeled cells were counted under a Leica DMI6000B microscope using a 20× objective and 1.6× magnification. The counting process was performed independently in two different areas of the two prepared slides until at least 50–100 cells per slide were detected and registered.

### Immunocytochemistry: Procedure and quantitative analysis

For immunocytochemistry, on irradiation days 3, 9, and 15, cells were seeded in technical duplicates on cover glasses in sterile non-coated six-well plates directly after treatment. After seeding, cell cultures were incubated for 24 h at +37°C and 5% CO_2_. All cells were fixed with 3% (w/v) PFA and 2% (w/v) sucrose in PBS for 10 min and permeabilized with 0.2% (v/v) Triton X-100 in PBS. Cells were incubated simultaneously with antibodies against Phospho (S139)-Histone H2AX (Millipore, clone JBW301) at a ratio of 1:200 and against 53BP1 (Bethyl Laboratories, #A300-272A) at a ratio of 1:400 in 2% (w/v) normal goat serum (NGS, Dianova) for 1 h. After several PBS washing steps, the cells were incubated simultaneously with Alexa Fluor anti-mouse IgG 488 or Alexa Fluor anti-rabbit IgG 546 (Invitrogen, both at a ratio of 1:250) for 45 min. The DNA was counterstained with DAPI (Invitrogen) and the cells were mounted with ProLong^®^ Gold (Invitrogen).

For quantitative analyses, residual foci were counted by two independent trained observers, using a Leica DMI6000B microscope with 63× objective and a 1.6× magnification. In order to detect foci in all three dimensions, the observer manually focused on each z-stack throughout the nucleus. The counting was performed independently in several different areas of slide until at least 50 cells were detected and registered. Every responsive cell (with one or more repair foci) was included in the evaluation.

### Senescence-associated beta-galactosidase activity

SA-β-gal staining was performed in MCF10A cells and fibroblasts at irradiation day 15 (for both irradiation regimens) after a cumulative dose of 40.05 Gy (HypoRT) or 30 Gy (NormRT) and on day 25 for NormRT (total dose 50 Gy), using the staining kit (Cell Signalling Technology) to detect the pH-specific (pH 6.0) activity of β-galactosidase, which is associated with senescence (22). The procedure was followed according to the manufacturer’s instructions. Briefly, cells were seeded in technical duplicates in a 24-well plate. After 20 h, cells were controlled to be attached and the development of blue color was documented 24 h after the fixation and staining procedure. Pictures in 24-well plates were taken with the staining solution remaining on the cells using the Nikon Eclipse TS100 inverse microscope. Quantification was performed using the Image J software. The number of senescent cells was normalized to the total cell number counted (up to 100 cells per well and at two positions).

### Colony formation assay

To determine the cell reproductive death after treatment with ionizing radiation, a modified clonogenic assay or colony formation assay (CFA) was performed. CFA is an *in vitro* cell survival assay based on the ability of a single cell to grow into a colony. The assay tests the ability of every cell in the population to undergo “unlimited” division, since only a fraction of seeded cells can produce colonies. Briefly, cells were seeded after irradiation at day 15 (for both irradiation regimens) after a cumulative dose of 40.05 Gy (HypoRT) or 30 Gy (NormRT) in six-well plates and at day 25 (NormRT only) after a total dose of 50 Gy in 12-well plates in technical triplicates. For each investigated cell line, a defined number of cells were seeded. At irradiation day 15 for both regimens, 500 cells/well for MCF10A, 750 cells/well for Bj5Ta, and 1250 cells/well and 1500 cells/well for HCC1397 and HCC1395, respectively, were seeded. At irradiation day 25 (NormRT only) 150 cells/well for MCF10A, 200 cells/well for Bj5Ta, and 400 cells/well and 500 cells/well for HCC1397 and HCC1395, respectively, were seeded. Untreated age-matched controls were seeded in parallel in technical triplicates in separate six-well plates: for MCF10A, 200 cells/well; for Bj5Ta, 250 cells/well; for HCC1937 and HCC1395, 500 and 750 cells/well, respectively. The medium was gently changed every 2 days. After *ca*. 7 days, incubation for MCF10A, 9 days for Bj5Ta, 12 days for HCC1937, and *ca*. 14 days of incubation for HCC1395, colonies were fixed with 3% (w/v) PFA and 2% (w/v) sucrose in PBS for 10 min, stained with 0.5% (w/v) crystal violet, and counted by microscopy. The plating efficiency (PE) as the ratio of the number of colonies to the number of cells seeded was estimated for each untreated cell line. Albeit not always, the more cells were seeded, the more plating efficiency was observed. The colony was defined to consist of at least 50 cells. The survival fraction (SF) of irradiated cells was expressed as a percentage of colonies per seeded cell after normalization by the plating efficiency of non-irradiated cells. Cell survival data was plotted as a logarithm of the SF versus dose.

### Statistical analysis

Statistical analysis was performed using GraphPad Prism (version 9.0.0; GraphPad Software). In order to compare differences between the two groups, a Student’s *t*-test was performed. Three or more groups were compared using one-way ANOVA (a repeated-measures analysis of variance). p Values below α < 0.05 were considered significant.

## Results

### Efficacy of hypofractionated and normofractionated irradiation regimens on the cell’s proliferation scale

**Figure 1:**
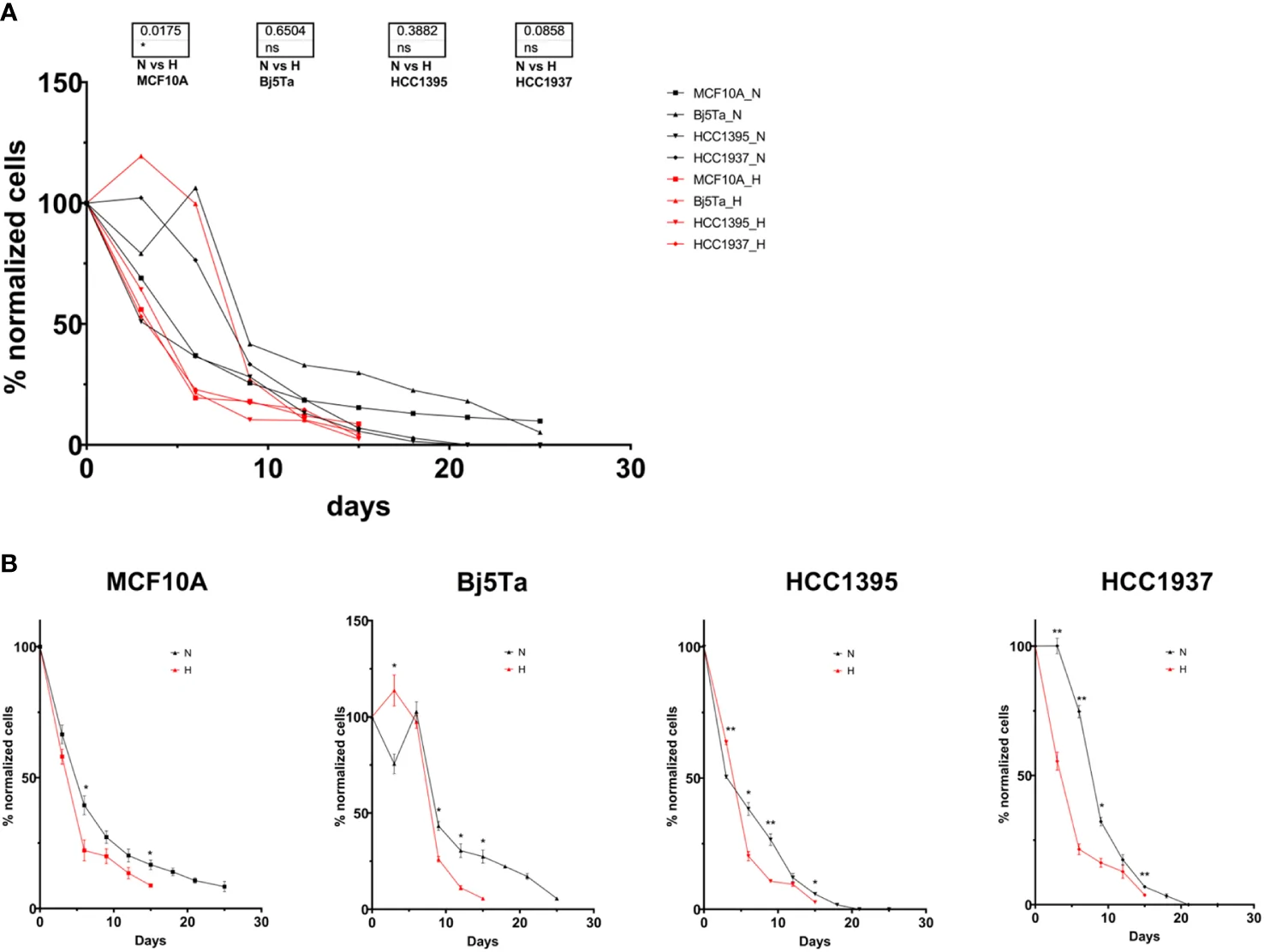
Cell proliferation capacity and growth rate during radiotherapy. The cell counts at specific irradiation days were normalized to the number of cells seeded at the start of the experiment and plotted as a percentage for all investigated cell lines **(A)**, or each cell line individually **(B)**. The red line and H represent HypoRT; the black line and N represent NormRT. *p<0.05, **p<0.005, n.s, non-significant.

We determined the proliferation capacity and growth rate of the employed cell cultures after fixed days of radiotherapy. The number of directly counted cells was continuously reducing over time. The growth rate of the Bj5Ta (HypoRT or NormRT) and HCC1937 (NormRT) were highest among all cell lines until day 9, but thereafter, all cells had almost equal proliferation (**Figure 1A**). There was a significant difference in the hypofractionated irradiation protocol compared to the conventional one for MCF10A cells and a nominally significant difference for HCC1937 cells (**Figure 1A**). There was also a difference between the cells lines (**Figure 1B**).

**Figure 2:**
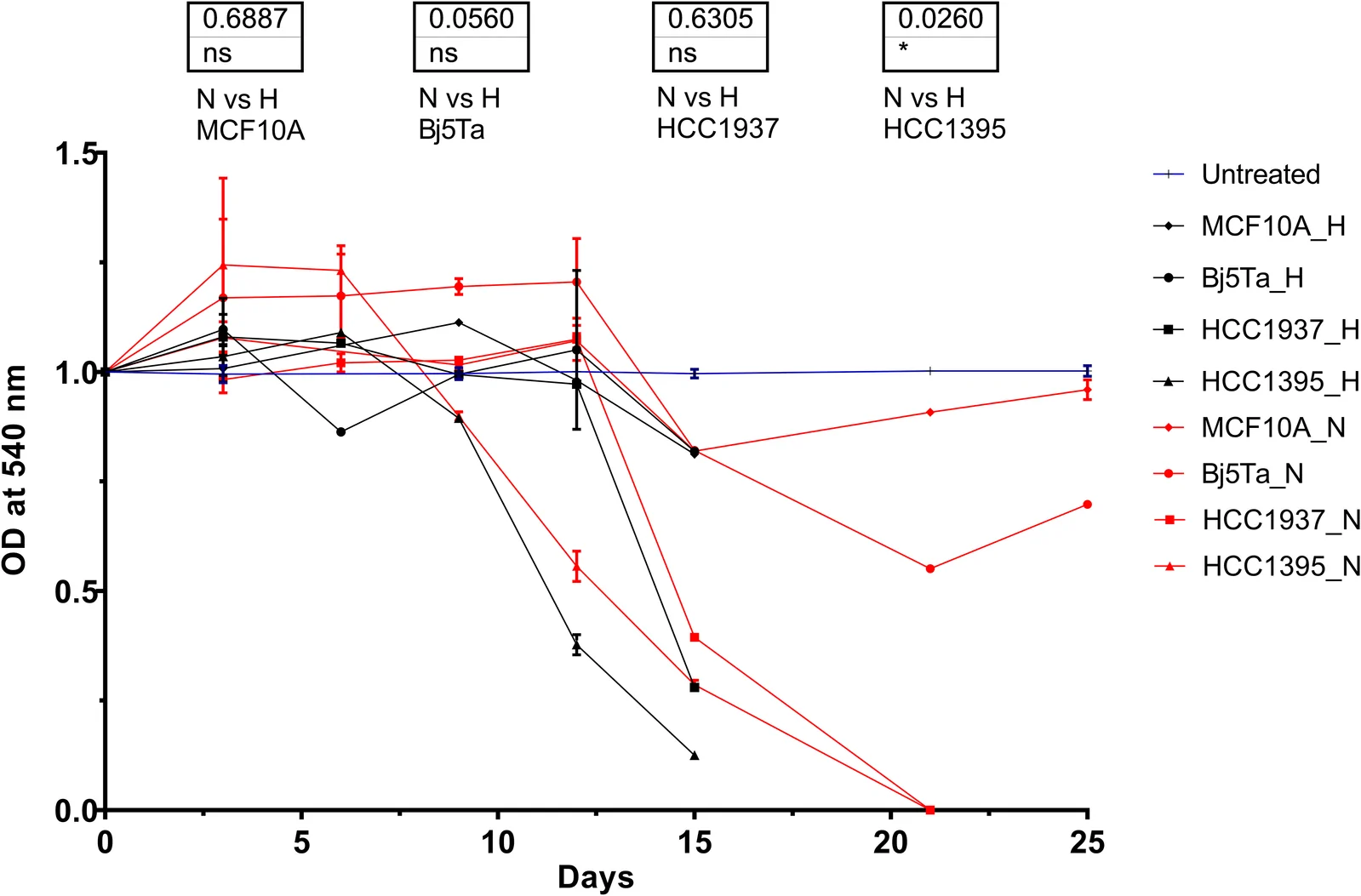
Cell growth rate during the radiotherapy evaluated in the MTT assay. OD values for each cell line were normalized to the appropriate values of untreated cells and plotted versus the irradiation day. The blue lines represent untreated values of the individual cell lines at specific experimental days, which were normalized to 1. The red lines correspond to the HypoRT protocol and black lines to the NormRT protocol, respectively. *p<0.05, n.s, non-significant.

By means of the MTT assay, we found a significant difference in growth rate and cell viability after the hypofractionated irradiation regimen, compared to the conventional one, for HCC1395 cells, and this observation was nominally significant for Bj5Ta cells (**Figure 2**).

**Figure 3:**
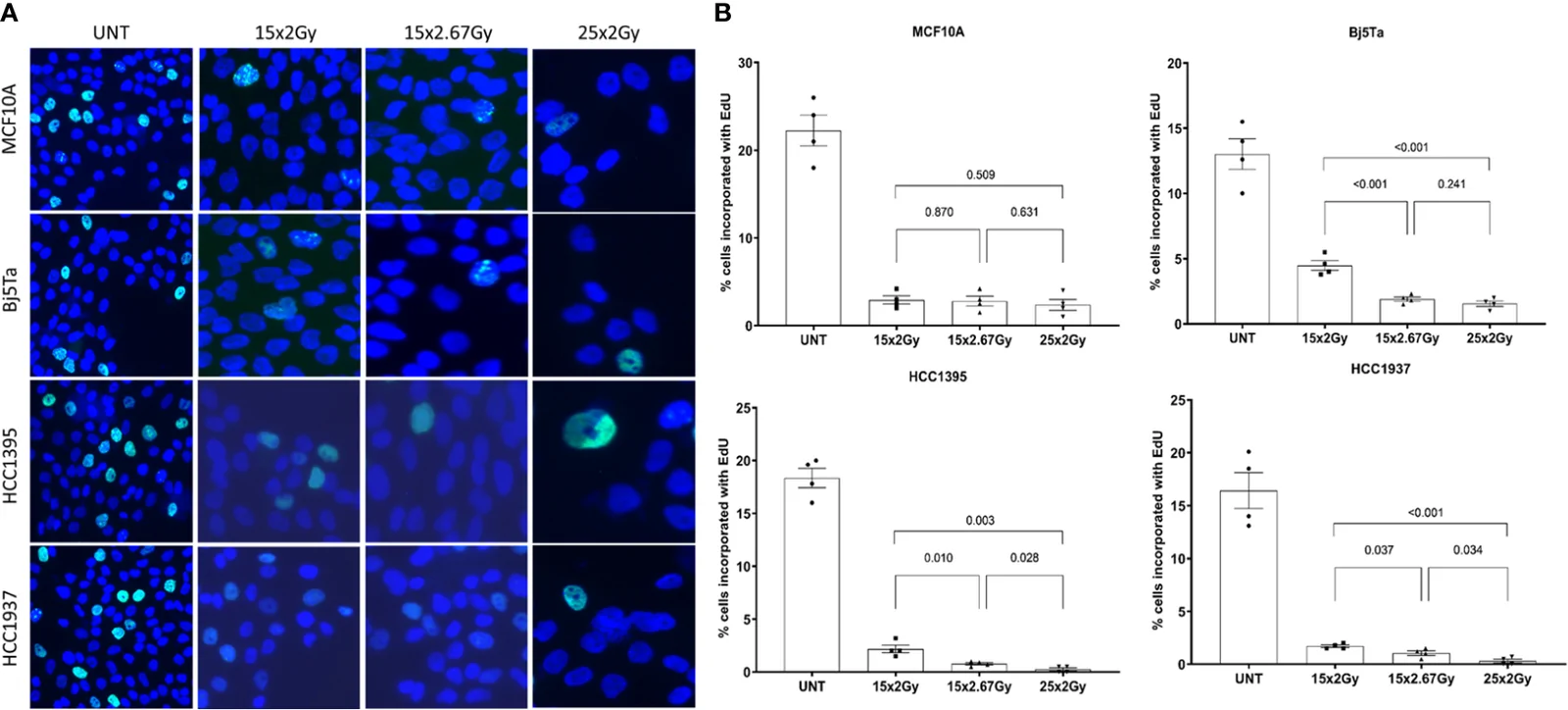
DNA synthesis-based cell proliferation evaluated on irradiation day 15 (both protocols) and on day 25 (NormRT only). Representative images of EdU incorporation staining **(A)** using conventional fluorescence microscopy (Leica DMI6000B) and evaluation **(B)**. The percentage of EdU-positive cells is presented as a bar plot +/- SEM.

In the DNA synthesis-based cell proliferation, we found the pronounced difference in both BC lines if the hypofractionated irradiation regimen was compared to the conventional one (**Figures 3 A, B**). However, the effect of decreased proliferation (newly synthesized DNA) was more significant for NormRT at day 25 contrary to HypoRT at day 15.

Normal cells (MCF10A and Bj5Ta) showed no difference after the total dose was applied, according to the irradiation regimen, although fibroblasts had a significantly reduced growth rate after HypoRT in contrast to NormRT at day 15 (**Figures 3A, B**).

The HCC1395 BC cell line was most sensitive to irradiation among all cell lines tested in proliferative assays, being more sensitive to the HypoRT regimen in the MTT assay (**Figures 1**, **2**). The HCC1395 and HCC1937 BC cell lines significantly slowed down proliferation at about irradiation day 15 (both irradiation protocols) and died after day 25. The MCF10A and Bj5Ta lines, being non-cancer cells, continued to grow (albeit at a slightly retarded rate) with daily exposure, and the total number on day 25 (NormRT) was 4.9 × 10^4^ for MCF10A cells and 4.7 × 10^4^ for Bj5Ta, respectively (started with 5 × 10^5^ and 9 × 10^5^ for MCF10A and Bj5Ta, respectively). At irradiation day 15, both lines had total cell numbers of 7.7 × 10^4^ and 2.7 × 10^5^ (NormRT) or 4.3 × 10^4^ and 4.7 × 10^4^ (last day of HypoRT) for MCF10A and Bj5Ta cells, respectively.

### Efficiency of DNA DSB repair after hypofractionated and conventional multifractionated radiotherapy

**Figure 4:**
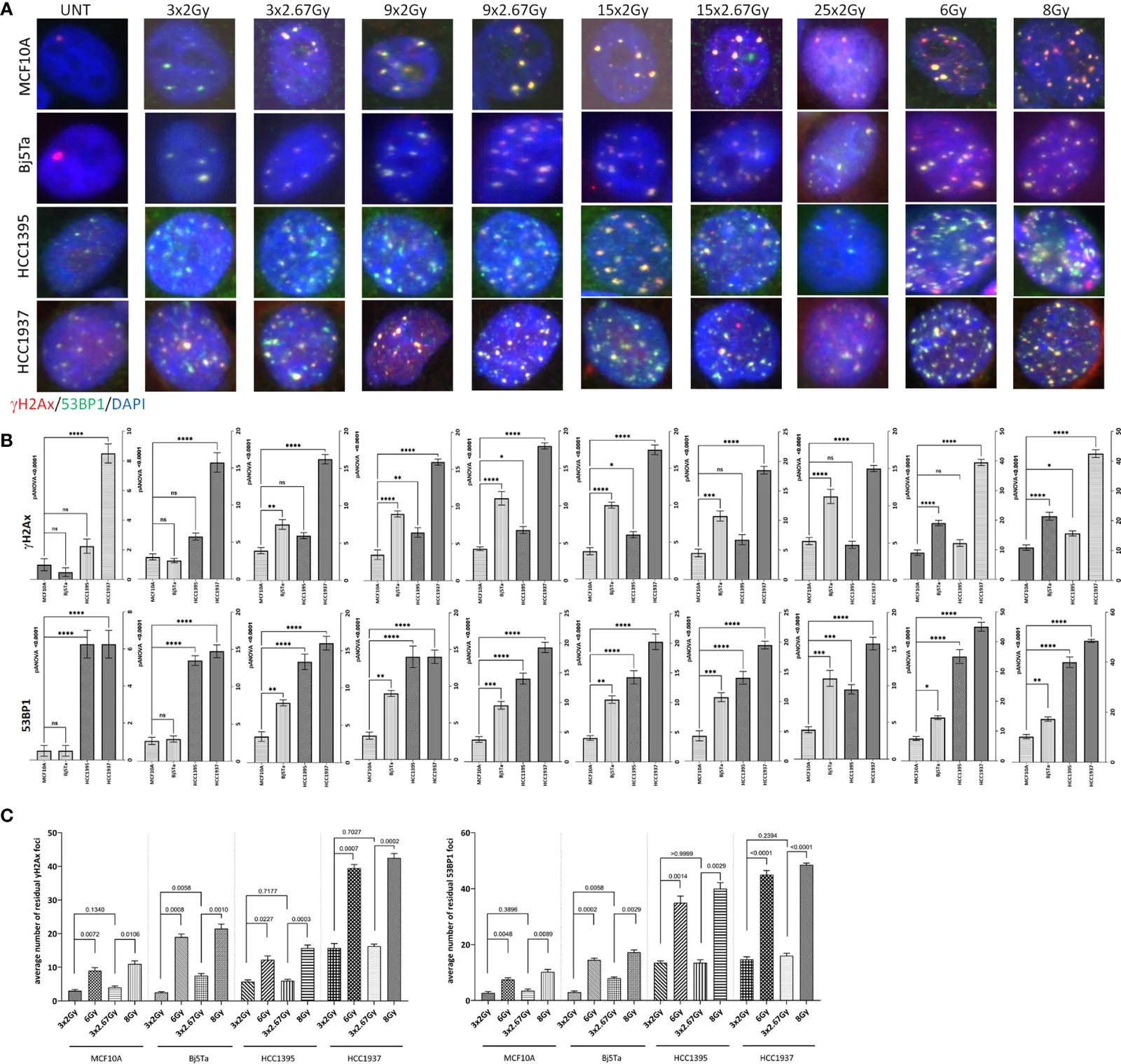
Immunocytochemical analysis of residual DNA damage foci after fractionated irradiation with the corresponding protocols. Representative images of residual γH2AX foci (red) and 53BP1 foci (green) double immunostaining **(A)** and evaluation **(B, C)** of γH2AX foci (top on B and left on C) and 53BP1 foci (bottom on B and right on C) 24 h after systematic irradiation with HypoRT or NormRT protocols at days 3, 9, 15, and 25 (NormRT only), using conventional fluorescence microscopy (Leica DMI6000B). DNA is counterstained with DAPI (UNT – untreated value “age-matched” to day 25). Evaluation data are presented as bar plots of average foci number (+/- SEM) per cell per slide from two slides. Values of 6 Gy and 8 Gy represent single-dose controls for fractionated irradiation with the NormRT or HypoRT protocol, respectively, at irradiation day 3. **(B)** Comparison of average residual DNA damage foci numbers between different cell lines and MCF10A cells. **(C)** Comparison of average residual DNA damage foci numbers (γH2AX and 53BP1) within the cell lines after fractionated irradiation (three fractions with respective protocols) and after a single dose of 6 Gy or 8 Gy, respectively. *p<0.05, **p<0.005, *** p<0.0005, **** p<0.0001, n.s, non-significant.

**Figure 5:**
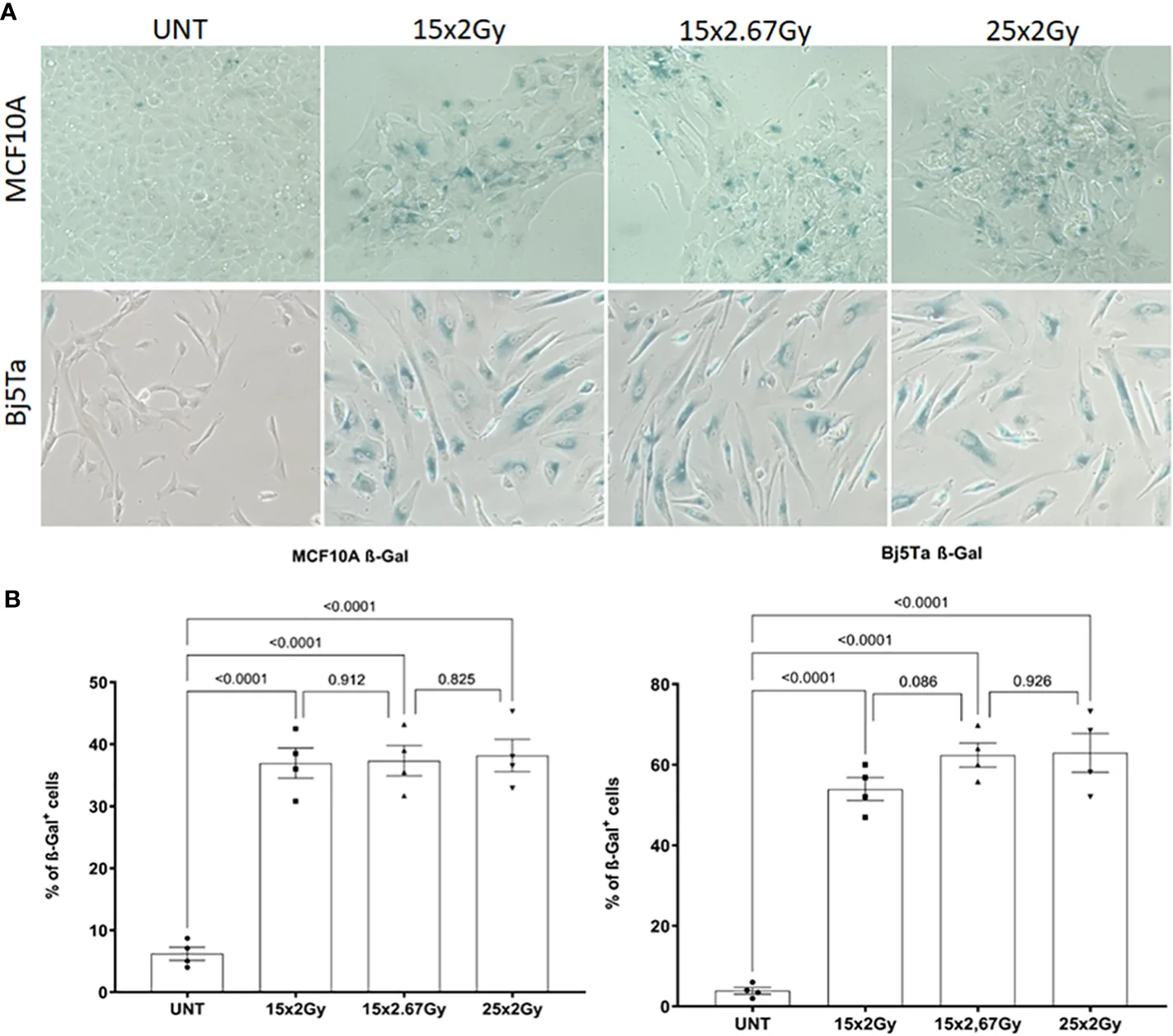
Senescence-associated beta-galactosidase (SA-βgal) activity analysis in MCF10A and Bj5Ta cells after fractionated irradiation with the corresponding protocols. Representative images of SA-βgal staining **(A)** and evaluation **(B)** of the percentage of senescent cells using inverse microscopy (Nikon Eclipse TS100 data from two technical replicates are presented as bar plots). Each dot represents a counting area (UNT – untreated “age-matched” to day 15).

To clarify the role of DNA damage response (DDR) proteins in cell survival after different regimens of radiotherapy, we analyzed residual γH2AX and 53BP1 foci in cells irradiated with the corresponding fractionated protocols. We also incorporated single-dose controls (6 Gy and 8 Gy) for irradiation day 3. We found that all of the tested cell lines had significantly lower numbers of residual γH2AX and 53BP1 foci after fractionated irradiation at day 3, than cells that had received the single dose (**Figures 4A, C**), suggesting that DNA repair could play a role in conferring cell survival after multiple fractions. There were clear differences between the BC cell lines with different mutational backgrounds, especially in contrast to the reference MCF10A cells (**Figures 4A, B**). However, HCC1395 had a higher ratio of 53BP1/H2AX foci and that was consistent with its known *NBN* mutation that impairs γH2AX accumulation after irradiation (21). From day 9 to day 15, we observed no significant increment in residual γH2AX and 53BP1 foci in all of the tested cell lines and from day 3 to day 9 only in fibroblasts for the NormRT regimen (**Table 1**). Since all employed cells had significantly elevated levels of residual foci (both types) after irradiation day 9 (**Table 1**), and persistent DNA damage foci may serve as a biomarker for cellular senescence, we next measured senescence-associated β-galactosidase activity in MCF10A cells and fibroblasts (20). The percentage of β-galactosidase positive cells was significantly increased in comparison to untreated state (**Figures 5A, B**), but there was no difference between different irradiation regimens. In addition, cells showed senescence-like phenotype also morphologically, with cellular hypertrophy, irregularities in shape, and vacuolization (**Figure 5A**), and these observations were true also for cancer cell lines.

**Table 1:** Evaluation of DNA DSB repair efficiency after hypofractionated and conventional multifractionated radiotherapy by means of residual foci.

Comparison values	MCF10A		Bj5Ta		HCC1395		HCC1937	
	γH2Ax	53BP1	γH2Ax	53BP1	γH2Ax	53BP1	γH2Ax	53BP1
UNT vs. 3×2 Gy	n.s	n.s	n.s	n.s	*	*	***	*
UNT vs. 3×2.67 Gy	n.s	n.s	***	***	*	*	***	*
UNT vs. 9×2 Gy	*	*	***	***	**	*	***	*
UNT vs. 9×2.67 Gy	*	*	***	***	*	*	***	*
UNT vs. 15×2 Gy	*	*	***	***	*	*	***	*
UNT vs. 15×2.67 Gy	*	*	***	***	**	*	***	*
UNT vs. 25×2 Gy	***	**	***	***	*	*	***	**
3×2 Gy vs. 3×2.67 Gy	n.s	n.s	**	**	n.s	n.s	n.s	n.s
3×2 Gy vs. 9×2 Gy	n.s	n.s	***	***	n.s	n.s	n.s	n.s
3×2 Gy vs. 15×2 Gy	n.s	n.s	***	***	n.s	n.s	n.s	n.s
3×2 Gy vs. 25×2 Gy	n.s	n.s	***	***	n.s	n.s	n.s	n.s
3×2 Gy vs. 6 Gy	**	*	***	***	*	***	***	***
3×2.67 Gy vs. 9×2.67 Gy	n.s	n.s	n.s	n.s	n.s	n.s	n.s	n.s
3×2.67 Gy vs. 15×2.67 Gy	n.s	n.s	n.s	n.s	n.s	n.s	n.s	n.s
3×2.67 Gy vs. 8 Gy	**	***	***	**	***	**	***	**
9×2 Gy vs. 9×2.67 Gy	n.s	n.s	n.s	n.s	n.s	n.s	n.s	n.s
9×2 Gy vs. 15×2 Gy	n.s	n.s	n.s	n.s	n.s	n.s	n.s	n.s
9×2 Gy vs. 25×2 Gy	n.s	n.s	n.s	n.s	n.s	n.s	n.s	n.s
9×2.67 Gy vs. 15×2.67 Gy	n.s	n.s	n.s	n.s	n.s	n.s	n.s	n.s
15×2 Gy vs. 15×2.67 Gy	n.s	n.s	n.s	n.s	n.s	n.s	n.s	n.s
15×2.67 Gy vs. 25×2 Gy	n.s	n.s	n.s	n.s	n.s	n.s	n.s	n.s

n.s, non-significant; *p < 0.05, **p < 0.01, ***p < 0.001.

Results of one-way ANOVA with multiple statistical test correction.

### Effects of hypofractionated and conventional irradiation regimens on survival of the cells

**Figure 6:**
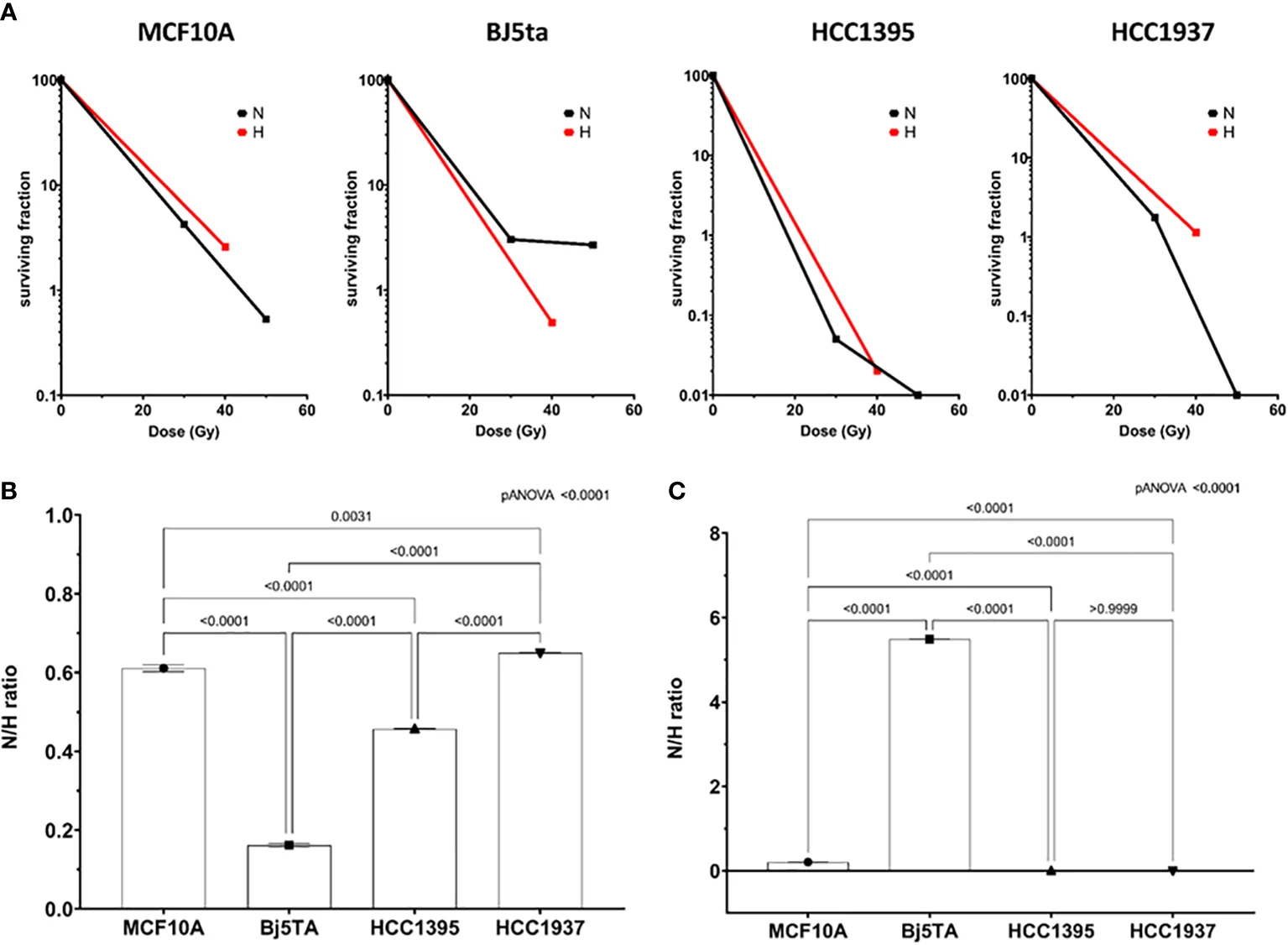
Survival after hypofractionated and conventional multifractionated radiotherapy. Clonogenic survival of the employed cell lines after irradiation with the corresponding multifractionated protocol **(A)**. The red line and H represent HypoRT, the black line and N represent NormRT. Non-irradiated cells were used as control for performing modified CFA. Efficacy of HypoRT versus NormRT radiotherapy at irradiation day 15 after a cumulative dose of 40.05 Gy (HypoRT) or 30 Gy (NormRT) **(B)**. The ratio of HypoRT to NormRT survival was calculated as follows: surviving fraction after 40.05 Gy/surviving fraction after 30 Gy. Efficacy of HypoRT versus NormRT radiotherapy by the end of both irradiation protocols. **(C)**. The ratio of HypoRT to NormRT survival was calculated as follows: surviving fraction after a cumulative dose of 40.05 Gy/surviving fraction after a cumulative dose of 50 Gy.

The plating efficiency in the performed CFA assay was lower for HCC1395 cells than for other cells (about 3% in HCC1395 compared with 23% in HCC1937 cells; about 53% in Bj5Ta cells and about 60% for MCF10A cells). The number of colonies in untreated cells tended to increase according to the increase of the number of cells seeded. This tendency was observed in all investigated cells, except HCC1395. After day 15 of irradiation, the HCC1395 cell line was most radiosensitive in a colony formation assay, especially for the HypoRT regimen, whereas HCC1937 cells had the same sensitivity to HypoRT or NormRT (**Figures 6A–C**). Bj5Ta and MCF10A cells tended to be also more sensitive to HypoRT vs NormRT, although after an appropriate cumulative dose for each regimen (40.05 Gy – HypoRT and 50 Gy – NormRT) Bj5Ta cells showed increased survival after NormRT regimen irradiation (**Figures 6A–C**). The responses of all investigated cells were different and could be distinguished from each other, indicating cell line specificity of effect.

## Discussion

Radiotherapy (RT) is an important component in the treatment of breast cancer, especially as an adjuvant approach in breast conserving therapy. Postoperative irradiation is also getting continuously important in the management of the TNBC subtype, although the benefit is still debatable, concerning overall survival (10, 11). Recent studies revealed no differences in dose fractionation adding an evidence to support the use of moderate hypofractionated whole-breast irradiation in TNBC patients (23, 24). The golden standard of care for many years was NormRT (with 45–50 Gy in 25–28 fractions), delivered with a long schedule over 5 weeks (9). In recent years, HypoRT (with 39–42.5 Gy in 13–16 fractions) is being established as a new standard. In several randomized trials, the similarity between effects after HypoRT and traditional 5-week NormRT has been shown (14, 20, 23–25) and HypoRT is now considered an accepted practice in numerous clinics, although many professionals remain skeptical and concerned about its efficiency and side effects. It is known that both tumor and normal cells generally can survive better when RT is delivered in fractions as compared to a single large dose. Thus, fractionated regimens may reduce damages to non-malignant cells, especially standard NormRT with a smaller dose per fraction, but this could also affect the anti-tumor efficacy in influencing the growth inhibition or metastatic potential and proliferation of malignancies.

Equal effectiveness and toxicities of HypoRT compared to NormRT for breast cancer have been proven in several randomized clinical trials since the early 2000s (14, 26–32). However, little is known so far about the cellular processes that take place during the medical radiation, applied over time in different-sized fractions, though understanding the mechanisms of side-effect occurrence, or those which promote cancer cell survival, could improve treatment and patient outcome and identify new strategies for more precise intervention. To our best knowledge, there are fewer studies providing some preclinical investigations on the *in vitro* radiobiological comparison of hypofractionation and conventional fractionation for any tumor type, mimicking the clinical situation. Most research studies in this field deal either with clinical trials and meta-analysis (14, 26–32) or with modeling radiobiological effects (33, 34). Regarding biological effects of exposure to ionizing radiation, most studies either utilize a single-irradiation dose or focus on fractionated irradiation, applying more fractions over a short time period, to establish surviving/resistant cell lines (35). Direct radiobiological comparison of fractionation regimens, for instance, in non-small cell lung cancer or glioblastoma cell models reflects the clinical situation with some advantages of hypofractionation for tumor control with no observed increase in radiotoxicity (36, 37). However, there is some evidence that hypofractionated radiotherapy can play a significant role in radioresistance and tumor recurrence and there is a need to optimize radiotherapy strategies, since different sites and types of tumors may respond differently to the same dose and fractionated irradiation (38). To address the question about the effectiveness and toxicity of HypoRT in comparison to NormRT on the cellular level in a triple-negative breast cancer model, we investigated how normal and tumor cells respond to differential regimens of radiotherapy in the clinical setting, using a combination of molecular and functional approaches.

Since irradiation can directly affect cells by triggering DSBs and inducing repair processes and other cellular effects, such as proliferation inhibition as well as cell death *via* apoptosis, necrosis, or senescence, we were investigating proliferation and growth capacity, efficiency of DNA DSB repair, and cell survival during HypoRT and NormRT irradiation regimens.

Different cell types as well as different cancer types and independent tumors of the same cancer type can have individual responses to ionizing radiation. Our results showed that investigated cells differ in their receptivity to different irradiation regimens. In general, the number of directly counted cells was continuously reducing over the irradiation time for both protocols. There was a significant difference in HypoRT protocol compared to NormRT for normal epithelial cells MCF10A. This difference was also nominally significant for HCC1937 BC cells. Another BC cell line, HCC1395, being the most sensitive in all approaches, exhibited significant difference in growth rate and cell viability after HypoRT compared to NormRT in MTT assay, and this observation was also nominally significant for Bj5Ta cells. We noticed unexpected higher absorbance values for all investigated cell lines in the first 12 irradiation days, independent from dose per fraction. If the absorbance values of the experimental samples are higher than the untreated control, this indicated an increase in growth rate/cell proliferation. Alternatively, if the absorbance rates of the experimental samples are lower than the untreated control; this indicated a reduction in the rate of cell proliferation or a reduction in overall cell viability. As observed in our settings, an increase in cell proliferation by means of MTT could also reflect the offset by cell death (i.e., apoptosis), which is more plausible. The different speed of reaction in the number of cells of the different cell types is caused by the inborn different turnover in tissue. Regular rhythm of mitosis and apoptosis is hardly changed by radiation of sublethal single doses and is fixed by the function of each cell: slower in glandular duct cells and faster in fibroblasts and also in tumor cells. In DNA synthesis-based cell proliferation, we found the difference of HypoRT compared to NormRT for both BC lines and the effect of decreased proliferation was more significant for NormRT at day 25 contrary to HypoRT at day 15. This observation fits with the clinical experience that the remission of a tumor can hardly be accelerated by the faster dose application during hypofractionation (39) and confirms clinical findings of different remission rates in irradiated tumors as well of other entities in clinical trials (39, 40). Normal cells showed no difference after the total dose was applied, according to the irradiation regimen, although fibroblasts had a reduced growth rate after HypoRT in contrast to NormRT at day 15. MCF10A, as mamma epithelial cells, are known to have a longer life span than the fibroblasts, and have a slower rate of radiation-induced apoptosis. This could only be accelerated by lethal single doses, but not by the sub-lethal dose of 2.67 Gy. As the number of cells with newly synthesized DNA in our study sinks slowly in MCF10A-cells after irradiation, the involution of glandular ducts develops months later than the remission of tumor cells. Thus, the quick remission in our cell culture of HCC1395 and HCC1937 is the same as shrinking tumors months before fibrosis occurred in patients.

The remaining tumor volume, which is persistent immediately after completion of HypoRT (still viable tumor cells in our settings), is in fact full of cells unable to undergo mitosis. Both BC cell lines after HypoRT formed some colonies in the CFA, but cells which were not taken in the experiment were further cultured in six-well plates under standard settings (without irradiation) and did not survive after day 25. This observation coincides with the clinical observation that the remission of the tumor is sometimes achieved even before the onset of normal tissue toxicity (40).

Clarifying the role of DNA DSBs repair in cell survival after different regimens of radiotherapy, we found that all of the tested cell lines had significantly lower numbers of residual γH2AX and 53BP1 foci after fractionated irradiation, than cells that had received the single higher dose. These results suggest that DNA repair could play a role in conferring cell survival after multiple fractions. The lower number of residual γH2AX and 53BP1 foci after fractionated radiotherapy explains the potential of higher single doses of radiotherapy to cause tissue necrosis (radionecrosis) as a late side effect despite equal effectiveness against the tumor (41). The functional status of DDR in general (and homologous recombination repair in particular) is known to be different in investigated cells and can be revealed only when cells are exposed to DNA damage. Indeed, our results support the notion that DNA damage repair occurred between radiation fractions in the first irradiation days, especially in normal MCF10A cells and fibroblasts (true only for the NormRT regimen). We noticed also increased values of residual foci in >comparison to the untreated state (especially for HypoRT regimen), which probably indicates that cells were unable to repair all DSBs before the next radiation dose induced new DNA damage. This observation was markedly significant in fibroblasts (**Table 1**), and in BC cell lines. From day 9 to day 15, we observed no significant increment in residual γH2AX and 53BP1 foci in all of the tested cell lines and, from day 3 to day 9, only in fibroblasts for NormRT regimen. These results suggest that either surviving cells adopted and have efficient DNA damage repair, or that replication stress, induced by irradiation and accumulation of DNA damage and DSBs, subsequently exceeded the repair capacity, which to some extent, reflects the proliferation scale observations. The majority of cells with irreparable DSB die of mitotic catastrophe, which reflects the inhibition of proliferation in our findings. Only adapted cells survive, with efficient DNA damage repair, as observed in the CFA assay for non-tumor cells.

All our results exemplify that the investigated cells differ in their receptivity and susceptibility to different irradiation regimens, and we could substantiate the already clinically proven equal effectiveness and toxicity of HypoRT for breast cancer compared to NormRT on a cellular level. This makes it easier to understand the differences in growth rate or the remission of tumor and development of fibrosis. Identifying the appropriate dosing scheme for any defined tumor entity may significantly impact on patient survival and therapy outcome.

## Conclusions

At the end of both therapy concepts (Normo and Hypo), normal and malignant cells reached almost the same endpoint of cell count and proliferation inhibition. BC cell lines significantly slowed down proliferation and died, whereas MCF10A and Bj5Ta lines, being non-cancer cells, continued to grow with daily exposure, although at a retarded pace. That confirms the clinical observations in the follow-up at the cellular level.

## Data availability statement

The original contributions presented in the study are included in the article/supplementary material. Further inquiries can be directed to the corresponding author.

## Author contributions

NB, HC, CH and RM contributed to conception and design and interpretation of data. SG, ML, and KK contributed to acquisition and interpretation of data, DR contributed to statistical analysis and interpretation of data; all authors were involved in drafting the manuscript, NB, HC, DR and RM have given final approval. All authors contributed to the article and approved the submitted version.

## Conflict of interest

The authors declare that the research was conducted in the absence of any commercial or financial relationships that could be construed as a potential conflict of interest.

## Publisher’s note

All claims expressed in this article are solely those of the authors and do not necessarily represent those of their affiliated organizations, or those of the publisher, the editors and the reviewers. Any product that may be evaluated in this article, or claim that may be made by its manufacturer, is not guaranteed or endorsed by the publisher.
